# Breast Cancer Metastasis to the Gastrointestinal Tract After Nine Years of Remission: A Case Report

**DOI:** 10.7759/cureus.104194

**Published:** 2026-02-24

**Authors:** Alex O Sophabmixay, Joshua C Obuch

**Affiliations:** 1 Gastroenterology, Philadelphia College of Osteopathic Medicine, Philadelphia, USA; 2 Gastroenterology, Geisinger Wyoming Valley Medical Center, Wilkes-Barre, USA; 3 Gastroenterology, Geisinger Commonwealth School of Medicine, Wilkes-Barre, USA

**Keywords:** biliary strictures, breast cancer metastasis, duodenal lesion, endoscopy duodenal biopsy, gastrointestinal metastasis

## Abstract

Gastrointestinal (GI) metastasis from breast cancer is rare and more commonly associated with invasive lobular carcinoma than invasive ductal carcinoma (IDC). Diagnosis of GI metastases secondary to breast cancer is difficult because patients may have long disease-free intervals or present with non-specific symptoms. We present a case of a 55-year-old patient with high-grade IDC in nine-year remission after bilateral mastectomy, axillary node dissection, reconstruction, and adjuvant chemoradiation who presented with recurrent abdominal pain and progressive, cholestatic liver enzyme elevation. Imaging and endoscopic evaluations demonstrated hepatic and duodenal lesions and biliary strictures. Biopsy confirmed the diagnosis of recurrent Stage IV IDC with metastasis to the duodenum, liver, lymph nodes, and spine. The patient received palliative systemic therapy and survived three years after recurrence diagnosis. Our case emphasizes that metastasis of IDC to the GI tract can occur after prolonged remission and that cholestatic liver enzyme elevation with new hepatic or biliary abnormalities should prompt consideration of metastatic recurrence in patients with a prior history of breast cancer.

## Introduction

In America, over 260,000 cases of breast cancer are diagnosed each year, making it the most common type of cancer in women, with over 40,000 deaths annually [1]. Two major types of breast cancer with high metastatic potential are invasive lobular carcinoma (ILC) and invasive ductal carcinoma (IDC), with 75-80% of the cases being IDC and 10-15% of cases being ILC [1]. Breast cancer recurrence can be local, regional, or distant, with distant recurrence representing metastatic spread to organs such as liver, lung, bone, or gastrointestinal (GI) sites [2,3]. Breast cancer metastasis to the GI tract is infrequent, occurring in approximately 10% of cases, and can be easily overlooked or misdiagnosed [3]. In cases of metastasis to the GI tract, the stomach, colon, and rectum are the most commonly affected sites, with the small intestine comprising only 19% of these cases [4]. Furthermore, the majority of breast cancers that metastasize to the GI tract are ILC, contributing to 64% of cases [5]. IDC rarely spreads to the GI tract, usually metastasizing to the liver, lungs, or bone [3]. Here, we report a case of a 55-year-old woman in remission after diagnosis of Stage II, Grade 3 IDC after bilateral mastectomies, who was found to have metastasis to the GI tract nine years later, which was diagnosed during evaluation and management of cholecystitis.

## Case presentation

**Figure 1:**
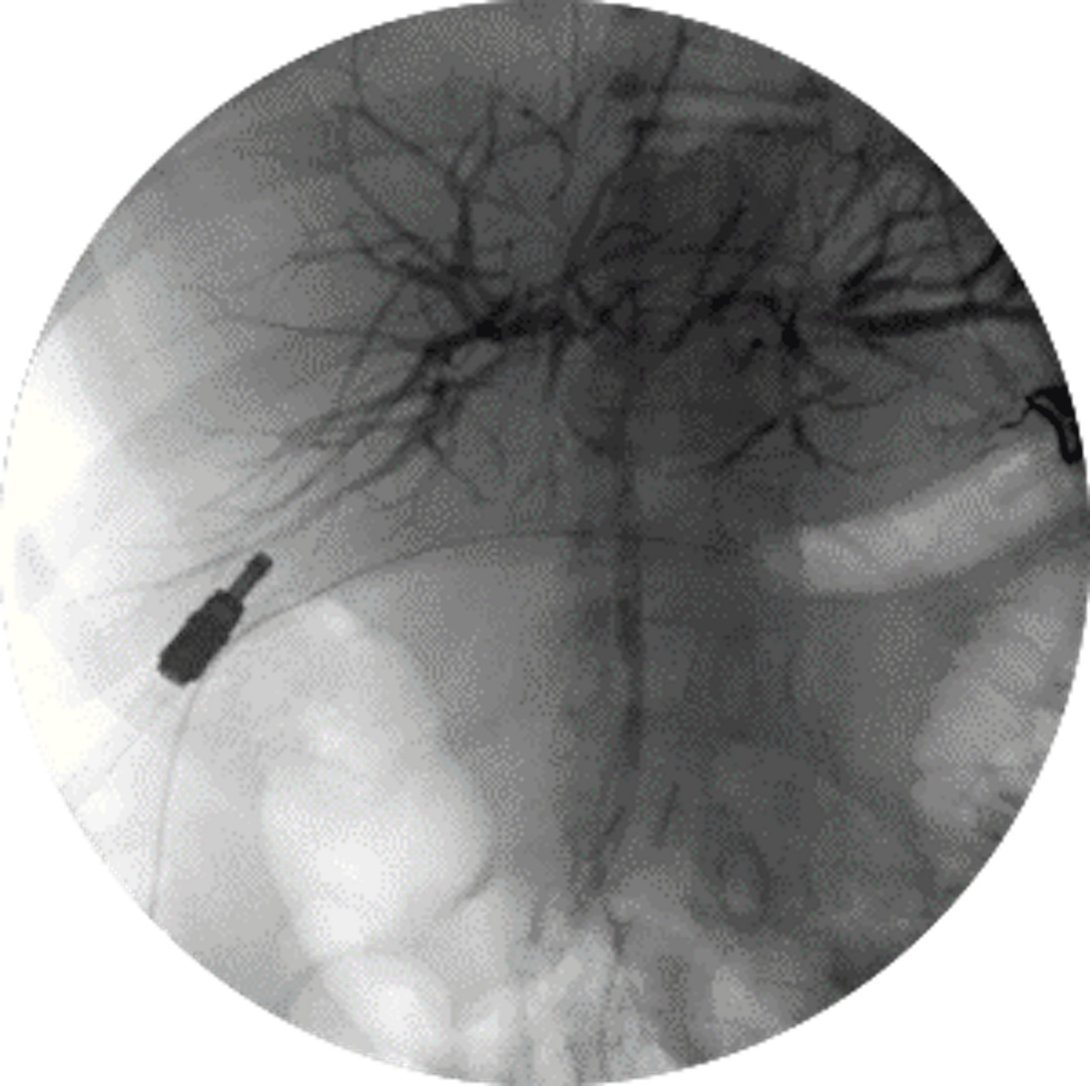
ERCP performed during the patient's first hospital admission for the management of cystic duct leak after laparoscopic subtotal cholecystectomy with drain placement. ERCP: Endoscopic Retrograde Cholangiopancreatography

In February of 2020, a 55-year-old postmenopausal woman initially presented to the emergency department and was subsequently hospitalized following several days of moderate epigastric pain radiating to the back. On evaluation, labs were significant for elevated liver enzymes, alkaline phosphatase (ALP), and lipase (Table 1). She denied jaundice, melena, hematochezia, hematemesis, nausea, vomiting, fevers, chills, or recent weight loss. She had a past medical history of Stage II, Grade 3, HER-2/Neu positive IDC of the left breast diagnosed in 2011, which was treated with left mastectomy, axillary lymph node dissection, and right prophylactic simple mastectomy with bilateral transverse rectus abdominis flap reconstruction, followed by adjuvant chemoradiation therapy. Imaging during the index emergency department visit and hospitalization, including right upper quadrant ultrasound and CT scan of the abdomen and pelvis, revealed concern for acute-on-chronic suppurative cholecystitis, for which she underwent laparoscopic subtotal cholecystectomy with drain placement followed by endoscopic retrograde cholangiopancreatography (ERCP) for management of cystic duct leak (Figure 1). Following the ERCP, she was discharged with an interval ERCP in three months for stent removal. However, 10 days after discharge from the index hospitalization, the patient returned with recurrent abdominal pain and was subsequently admitted for a second time. Liver enzymes, ALP, and lipase were all elevated. However, alanine aminotransferase, ALP, and lipase were markedly elevated over the index admission (Table 1).

**Table 1:** Comparison of liver enzyme and lipase laboratory values between the patient's first and second hospital admissions.

	First Admission	Second Admission	Reference Range (U/l)
Aspartate Aminotransferase (U/l)	379	286	10-35
Alanine Aminotransferase (U/l)	475	611	10-35
Alkaline Phosphatase (U/l)	210	480	35-130
Lipase (U/l)	127	200	13-60

**Figure 2:**
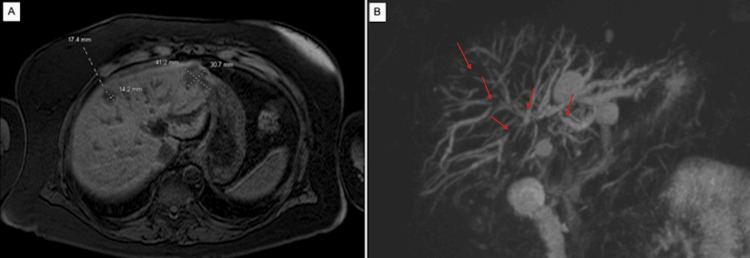
MRI (A) and MRCP (B) performed during the second hospital admission demonstrating hepatic lesions and intrahepatic biliary strictures, respectively. Red arrows indicate representative intrahepatic biliary strictures with associated upstream ductal dilation. MRI: Magnetic Resonance Imaging; MRCP: Magnetic Resonance Cholangiopancreatography

**Figure 3:**
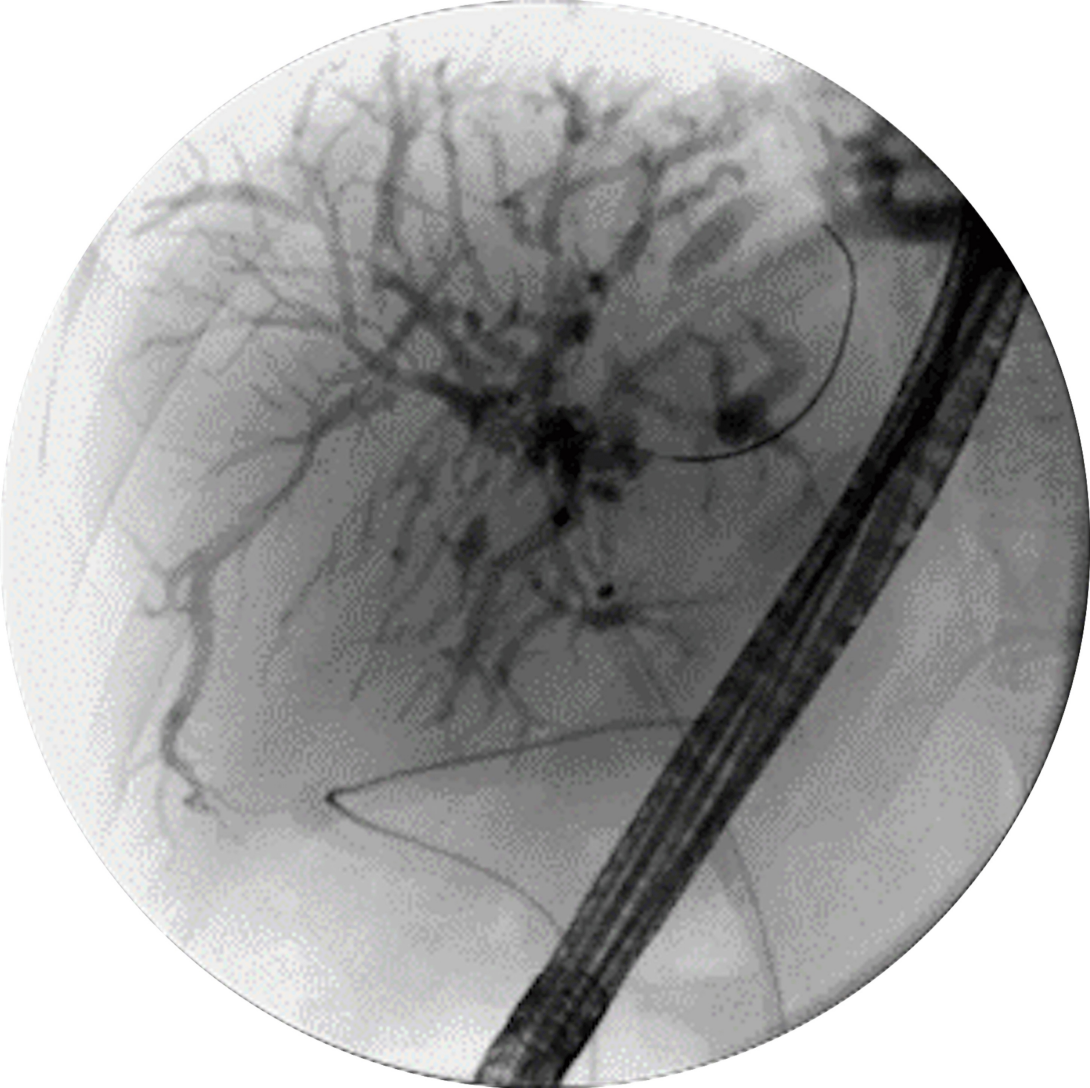
ERCP performed during the second hospital admission, 10 days after discharge, demonstrating multiple segmental and severe biliary strictures in the right and left hepatic ducts and all intrahepatic branches. ERCP: Endoscopic Retrograde Cholangiopancreatography

**Figure 4:**
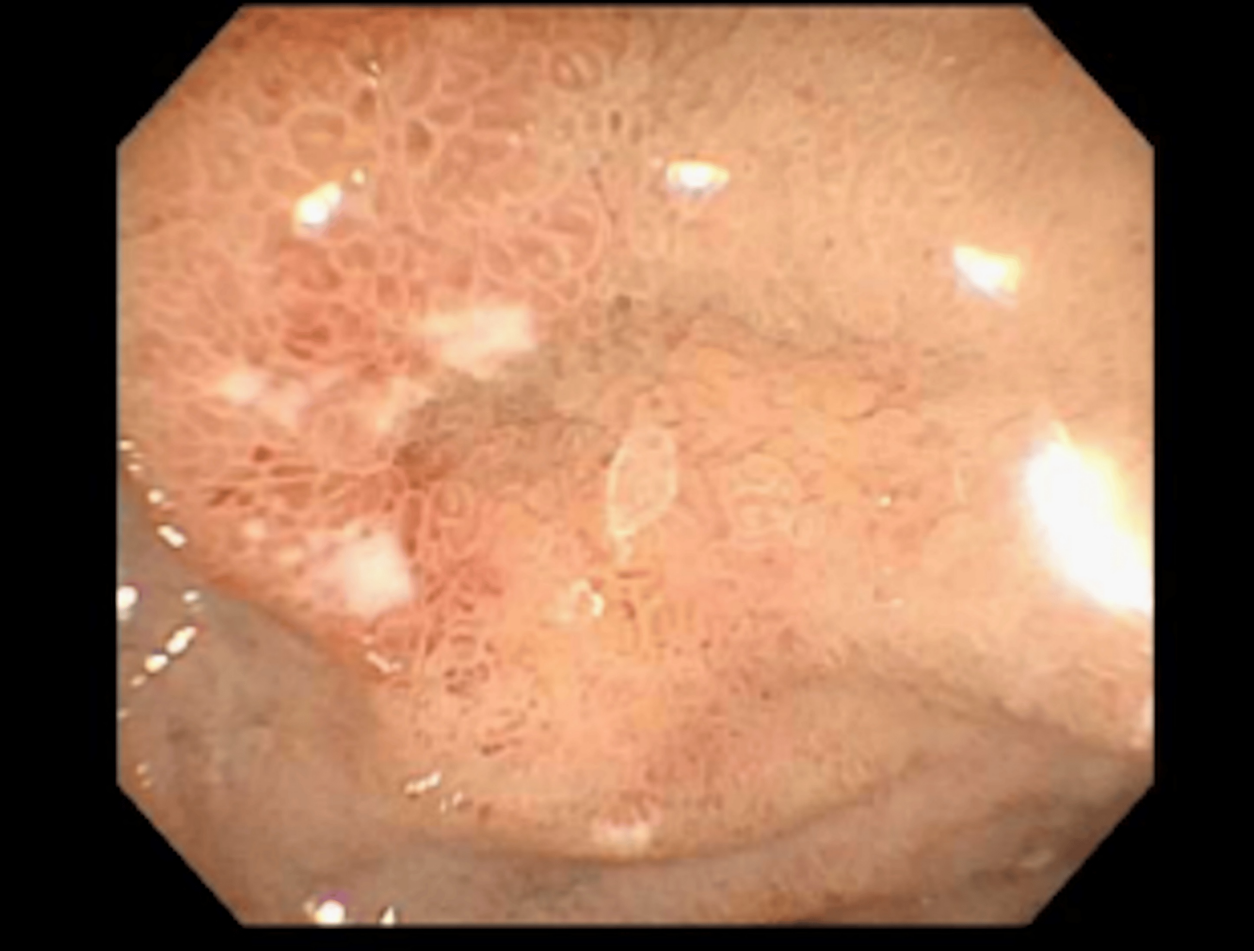
Image of the biopsied duodenal erosion captured during endoscopy.

A repeat CT scan of the abdomen with contrast showed new poorly defined hypodensities in the liver, prompting an abdominal MRI that showed two hepatic lesions concerning for abscesses and a magnetic resonance cholangiopancreatography revealing intrahepatic biliary strictures with associated upstream dilation and several small septated hepatic cysts communicating with the biliary tree (Figure 2). The CA 19-9 tumor marker was unremarkable. A repeat ERCP was performed, which showed multiple segmental and severe biliary strictures in the left and right hepatic ducts and all intrahepatic branches (Figure 3). During endoscopic evaluation, duodenal erosions were noted and biopsies were performed (Figure 4). Brushings for cytology were negative for malignancy. Dilation of the right intrahepatic stricture was performed. Wire access and stenting across the left intrahepatic strictures were unsuccessful. Thus, the patient underwent left-sided percutaneous transhepatic biliary drainage by interventional radiology. 

**Figure 5:**
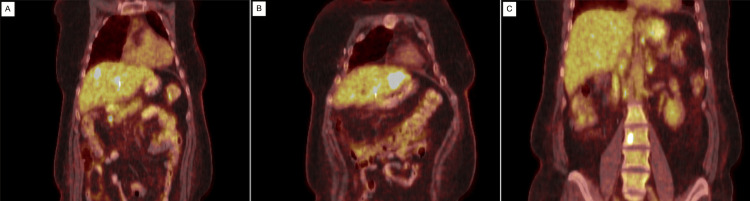
Full-body PET CT scan performed during the second hospital admission demonstrating multiple hypermetabolic lymph nodes in the chest and abdomen (A), as well as multiple hypermetabolic hepatic (B) and spinal (C) lesions. PET: Positron Emission Tomography; CT: Computed Tomography

Duodenal biopsies suggested metastatic breast carcinoma with positive HER-2 oncoprotein expression. Due to the presence of strictures in the intra- and extrahepatic biliary tracts, the patient underwent a core needle biopsy of the liver which also suggested metastatic breast carcinoma. A subsequent full-body PET scan was notable for multiple hypermetabolic lymph nodes in the chest and abdomen, as well as multiple hypermetabolic hepatic and spinal lesions consistent with multifocal metastatic disease (Figure 5). The patient was diagnosed with recurrent Stage IV IDC of the left breast with widespread metastatic disease to the liver, duodenum, lymph nodes, and bones. She was started on palliative systemic therapy with docetaxel, trastuzumab, and pertuzumab given once every three weeks for a total of six cycles, followed by the continuation of trastuzumab and pertuzumab. However, she ultimately passed from her disease process three years after recurrence diagnosis.

## Discussion

Metastases of breast cancer, especially IDC, to the GI tract are rare and often overlooked [6,7]. The majority of breast cancer cases that do metastasize to the GI tract (i.e., stomach, small intestine, colon, rectum) are from ILC [5]. The high rate of ILC metastasis to the GI tract is thought to be due to the small size and shape of ILC cells, allowing them to spread hematogenously to well-vascularized areas of the GI tract like the small intestine and stomach [8]. In addition, loss of E-cadherin protein expression leads to discohesiveness between lobular cells, promoting dissemination, which allows for migration to areas with microanatomy that provide an environment conducive for growth and survival such as the GI tract, ovaries, and peritoneum [9,10]. Very few cases of delayed metastasis for IDC have been reported, and the exact mechanisms of disease recurrence are unclear [5]. However, metastatic recurrence years after definitive therapy is plausible and is thought to be a consequence of minimal residual disease, in which disseminated tumor cells seeded prior to definitive treatment persist in a dormant state until they reactivate [11].

There are multiple reasons why the diagnosis of GI metastases secondary to breast cancer is difficult. Patients can have long disease-free intervals, such as our patient, who was disease-free for nine years. They can also have diverse and non-specific symptoms at presentation such as nausea, vomiting, dysphagia, weight loss, tenesmus, abdominal obstruction, and abdominal discomfort, which can all be attributed to other GI pathologies such as primary gastric carcinoma, colorectal cancer, inflammatory bowel disease, and other mechanical and vascular etiologies [5]. This case highlights that cholestatic liver enzyme elevation, particularly when accompanied by new liver lesions and intrahepatic biliary strictures, should raise consideration of metastatic disease in patients with a history of breast cancer presenting with nonspecific abdominal symptoms.

Endoscopic findings concerning breast cancer metastasis usually show large ulcers, polyps, diffuse infiltration, or external compression [12]. However, endoscopy alone may not be adequate for proper diagnosis [13]. In this patient, small duodenal erosions were the only pertinent luminal findings, emphasizing the role of having a low threshold to biopsy any atypical findings on endoscopy in patients with a breast cancer history. Endoscopic biopsies might be negative, especially in the early stages of metastasis since early spread initially involves the submucosal layer, making superficial biopsies inconclusive [9]. Deep biopsies might improve diagnostic accuracy [5]. In the late stages of metastasis, invasion of the entire bowel wall can manifest as an ulcer and signs of GI bleeding, such as melena or hematemesis [14]. Endoscopic ultrasound may serve as an adjunctive modality in similar cases by allowing for evaluation and sampling of submucosal lesions that are not detected on standard endoscopy or imaging. 

Immunohistochemical staining is essential in differentiating metastatic disease to the GI tract from primary GI malignancy [3]. In this case, duodenal pathology was positive for AE1/3, CAM 5.2, and GATA-3 markers, which suggested breast carcinoma metastasis [15-17]. Likewise, the liver core needle biopsy stained positive for GATA-3 and CK7, which are used for diagnosing breast carcinoma [18]. These findings confirmed that the tumor was due to metastatic disease and not primary malignancy in the GI tract. 

Our patient was diagnosed with recurrent Stage IV IDC of the breast because she presented to the emergency department with symptoms of cholecystitis, which led to findings of biliary strictures noted on imaging and further workup with ERCP, biopsy sampling, and immunohistochemical staining. Once GI metastasis was confirmed, systemic evaluation was pursued to identify other sites of metastasis, which included the liver, lymph nodes, and spine. Breast cancer survivorship guidelines recommend against routine screening for metastases in asymptomatic patients [19]. Therefore, breast cancer metastases to the GI tract are typically detected in a symptom-driven manner. Circulating tumor DNA assays may detect molecular residual disease and identify recurrence before clinical manifestation, but they are not part of standard clinical practice yet [20].

## Conclusions

This case highlights that GI metastasis from invasive ductal breast cancer, although rare, can occur after a prolonged disease-free interval and present with nonspecific GI or biliary manifestations. Clinicians should maintain a high index of suspicion for metastatic recurrence in patients with a remote history of breast cancer and pursue timely tissue diagnosis with immunohistochemical confirmation. Although surveillance for metastatic breast cancer in asymptomatic patients is not recommended, emerging tools such as circulating tumor DNA assays may assist in earlier detection and monitoring of recurrence, but their role in standard clinical practice remains under investigation.
